# Guided SEFFI and CaHA: A Retrospective Observational Study of an Innovative Protocol for Regenerative Aesthetics

**DOI:** 10.3390/jcm13154381

**Published:** 2024-07-26

**Authors:** Fabrizio Melfa, Alec McCarthy, Shino Bay Aguilera, Jani van Loghem, Alessandro Gennai

**Affiliations:** 1 Mediaging Clinic Center, 90143 Palermo, Italy; dottormelfa@gmail.com; 2 Merz Aesthetics, Raleigh, NC 27615, USA; alec.mccarthy@merz.com; 3 Shino Bay Cosmetic Dermatology & Laser Institute, Fort Lauderdale, FL 33301, USA; 4 UMA Academy, 1017 VV Amsterdam, The Netherlands; 5 Studio Gennai, 40128 Bologna, Italy

**Keywords:** SEFFI, SVF, CaHA, calcium hydroxylapatite, Radiesse, skin priming, stromal vascular fraction, fat grafting

## Abstract

**Background/Objectives**: This retrospective observational study sought to determine the efficacy and safety of an innovative combined treatment protocol using guided Superficial Enhanced Fluid Fat Injection (SEFFI) and calcium hydroxylapatite (CaHA) in facial rejuvenation. **Methods**: A total of 158 patients (149 females and 9 males) underwent the combined treatment of guided SEFFI and diluted/hyperdiluted CaHA. The study evaluated treatment outcomes at 30, 90, and 150 days post-treatment using the Global Aesthetic Improvement Scale (GAIS) and three-dimensional photogrammetric analysis. **Results**: The combined treatment demonstrated consistent enhancement in skin quality and facial volume across temporal, malar, zygomatic, and jawline regions. At 90 days post-treatment, substantial improvements were observed, with the GAIS scores reflecting significant enhancements in both skin quality and volume, which were sustained or slightly improved by 150 days. Minor complications, predominantly ecchymosis at the injection sites, resolved within a week, confirming the treatments’ safety. **Conclusions**: The integration of guided SEFFI and CaHA resulted in significant improvements in skin quality and facial volume with minimal complications. Further research is recommended to consolidate these findings and explore long-term outcomes.

## 1. Introduction

As we age, our skin undergoes progressive aging marked by numerous physiological and structural changes [1]. Skin aging is influenced by both intrinsic and extrinsic factors. While intrinsic aging is a natural, genetically driven process, extrinsic aging arises from external influences like UV radiation, pollution, and lifestyle choices [2,3,4]. A primary factor in both intrinsic and extrinsic aging is the decline in the Extracellular Matrix (ECM), a vital network of macromolecules essential for skin structure and function [5]. Apart from changes in the ECM, changes in cellular activity and communication influence tissue aging. Aging tissue also shows reductions in tissue perfusion. In a regenerative procedure, angiogenesis is essential to provide the newly formed and activated tissue with essential micronutrients as well as oxygen [3,4,5].

Key components of the ECM, such as collagen, elastin, fibronectin, and hyaluronic acid, have distinct roles in skin health [6]. Collagen provides structural support, elastin ensures elasticity, fibronectin aids in ECM regeneration, and hyaluronic acid maintains hydration [7]. Alterations in these components influence skin texture, firmness, and overall appearance [8]. Metalloproteinases, enzymes integral to ECM balance, are directly involved in skin aging. In youthful skin, metalloproteinases (MMPs) are well regulated and preserve the ECM’s integrity. However, aging disrupts the balance between MMPs and their inhibitors, leading to excessive MMP activity, which in turn degrades the ECM, causing wrinkles, sagging, and other aging signs [9].

In esthetic medicine, calcium hydroxylapatite (CaHA) (Radiesse^®^, Merz Aesthetics, Frankfurt, Germany) has become a valuable tool for facial rejuvenation. In addition to its dual properties of direct filling and biostimulation, CaHA has been shown to downregulate degradative MMP activity [10,11]. By physically occupying space as a result of its carrier gel, CaHA can immediately restore facial volume, while also stimulating the body’s natural collagen, elastin, proteoglycan, and neovascularization with the CaHA microsphere mechanotransduction of fibroblasts [7,12,13,14].

Adipose tissue, rich in mesenchymal stem cells (MSCs), has garnered attention for its regenerative potential, especially with Stromal Vascular Fraction (SVF) [15]. SVF, a diverse cell mixture that can be obtained from adipose tissue, offers a wealth of regenerative potential that can be used for skin rejuvenation and tissue regeneration [16]. These cells aid in skin regeneration, produce vital ECM components, and have anti-inflammatory properties beneficial for skin quality [17,18,19]. The Superficial Enhanced Fluid Fat Injection (SEFFI) technique is a novel method for harvesting and administering SVF for regenerative esthetics [20]. This technique allows healthcare providers to non-surgically collect superficial adipose tissue. SEFFI offers a tailored, minimally invasive approach to anti-aging skin therapy, tapping into the regenerative potential of a patient’s adipose tissue [21]. Guided SEFFI offers a minimally invasive approach to anti-aging that leverages the regenerative potential of the patient’s own adipose tissue [21,22,23].

Clinicians are now exploring the synergy between biological and material-mediated regenerative effects and have introduced the concept of skin priming [24]. This technique involves diluting or concurrently delivering CaHA with therapeutic diluents. For instance, studies have combined CaHA with micronutrient mesotherapy, hyaluronic acid, platelet-rich fibrin, and exosomes [24,25,26,27]. Gennai et al. introduced the concept of priming CaHA with micro-fragmented fatty tissue with promising results [22]. Following this, Melfa et al. conducted a study combining fragmented fatty tissue with CaHA to observe the biological effect of the combination [28]. The study demonstrated that cells differentiate towards mesenchymal lineages expressing mesenchymal markers by flow cytometry analysis. Combining emulsified fat tissue prepared with guided SEFFI technique with CaHA products can counteract the loss of volume and skin aging. 

Considering these findings, the authors have developed a protocol combining adipose tissue grafts from the guided SEFFI technique with CaHA for regenerative treatments. However, no study has concurrently delivered autologous adipose tissue and CaHA biostimulator microspheres in clinical practice. Based on the observed synergisms in our previous ex vivo and in vitro studies, clinical correlates of the efficacy of a combined treatment were hypothesized [28]. The efficacy and safety of such a treatment have not yet been elucidated either. Therefore, this article sought to gather the safety and efficacy results of over 150 cases combining guided SEFFI and CaHA for anti-aging treatments.

## 2. Materials and Methods

We conducted a retrospective observational study combining the use of CaHA injections in conjunction with the guided SEFFI procedure, which was performed using the disposable all-in-one medical device SEFFILLER™ (manufactured by SEFFILINE srl, Bologna, Italy). The study was conducted in private medical facilities affiliated with some of the authors, and it encompassed patients treated between January 2021 and March 2023. A total of 158 consecutive patients presenting with signs of facial aging were included in the study. 

### 2.1. Inclusion Criteria

Eligible participants for this study were healthy individuals aged between 40 and 65 years with stable Body Mass Indexes (BMI) between 18 and 25. Furthermore, they had not received any facial cosmetic medical or surgical interventions in the five months leading up to their treatment. The targeted treatment areas included the temporal, malar, zygomatic, and jawline regions. Prior to treatment, all the participants signed informed consent for the treatment and documentation of their data, thus following the guidelines stipulated in the Declaration of Helsinki. They granted permission for the utilization of their data, images, and subsequent analyses for academic research.

### 2.2. Guided SEFFI Preparation

**Figure 1:**
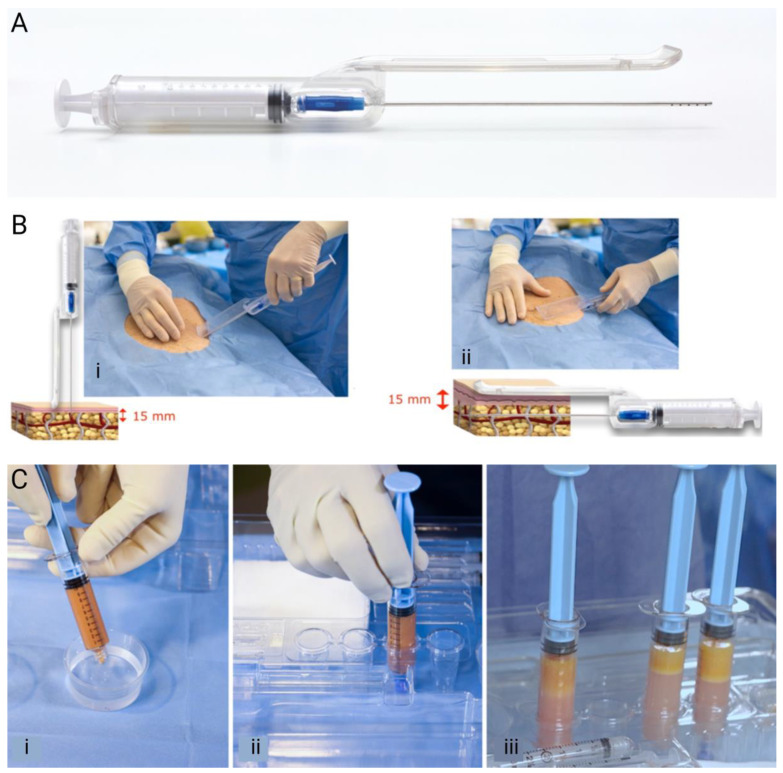
Adipose tissue harvesting using guided SEFFI. (**A**) The SEFFI guide assembled with the syringe and cannula. (**B**) The extraction of the adipose with (**i**) the cannula introduction perpendicular to the skin through a port from an 18G needle; (**ii**) the rotation of the guide 90° and the introduction of the cannula in the superficial subcutaneous plane for adipose extraction. (**C**) Harvesting the removed tissue by (**i**) rinsing with saline, (**ii**) closing the syringe with a Luer lock cap, and (**iii**) allowing the layers to separate. (**A**,**B**) are reprinted with permission from Gennai et al. 2023 [22]. (**C**) is reprinted with permission under CCBY from Melfa et al. 2023 [28].

Guided SEFFI procedures were performed following the device’s user manual and with the patients under local anesthesia. Adipose tissue was harvested from either the abdomen or the trochanteric region using a 10 mL syringe equipped with a microperforated side-port cannula inserted into the patented guide (side-port holes 800 microns) (Figure 1A) [21,22]. Gentle back-and-forth fanning movements during aspiration with the guide ensured tissue collection from the superficial adipose tissue (SAT) adjacent to the dermis (Figure 1B(i,ii)). Once 5 mL of tissue was obtained, it was rinsed with saline and allowed to stratify into two layers by gravity (Figure 1C(i,ii)). After a few minutes, the washing liquid was discharged, and the tissue was prepared for injection (Figure 1C(iii)).

The fresh micro-fragmented adipose tissue obtained with the guided SEFFI technique does not require further manipulation due to the small dimensions of the harvested clusters ensured by the SEFFI cannula. This is ideal, as Cucchianai and Corrales have shown that any substantial manipulation, aggressive harvesting, and ambient air exposure reduce the viability and stemness of the tissue [23,29]. Only light manipulation (passaging from one syringe to another) should be performed if the viscosity is to be diluted, such as for delivery into the delicate areas of the face. 

Facial areas have different skin and subcutaneous thicknesses, and it is important to inject the proper fluidity of filler when injecting superficially to reduce the risk of visibility and lumpiness [30]. In cases where decreased viscosity was desired, a further reduction in fat cluster dimensions was carried out using an emulsion procedure. Two 10 mL syringes were connected through a transfer provided in the guided SEFFI device and based on the desired size of the adipose tissue clusters, a designated number of passages (transfers back and forth between syringes) was carried out (Table 1). The entry point of the collecting area was covered with Steristrip^®^ and a mild compression was applied with kinetic tape dressing. 

### 2.3. CaHA Preparation

The authors used both diluted (1:1) and hyperdiluted (1:>1) CaHA based on the global consensus guidelines [31]. Briefly, the 1.5 mL filler syringe was connected and transferred to a 3 mL syringe via a luer lock transfer. Then, the 3 mL syringe containing the undiluted CaHA was connected to a 3 mL syringe containing 1.5 mL of saline solution (diluted 1:1) and homogenized by passing from one syringe to the other. For hyperdilutions, the filler was transferred to a 5 mL syringe and connected to another 5 mL syringe containing 3 mL of saline solution (hyperdiluted 1:2) and homogenized with back-and-forth passage between the syringes.

### 2.4. Facial Injection Procedure

Prior to injection, the patients were evaluated and marked for treatment while standing but were positioned supine for the procedure. Local anesthesia was administrated at the cannula entry points. The procedure started by injecting diluted or hyperdiluted CaHA by cannula in the subcutaneous plane using a linear retrograde fanning technique. The authors used a 1:1 dilution for the facial areas with thicker skin and the hyperdiluted formula (1:2) for the areas with thinner skin. At the end of the CaHA injection, a gentle massage was performed to optimize the spread of the CaHA microspheres [32]. Following CaHA injection, the adipose tissue (600 µm lobules; emulsified with 3 passes) was injected with either a 20- or 21-gauge cannula in the subcutaneous superficial plane using a linear retrograde fanning technique. In the areas with thinner skin, the adipose tissue with cluster diameters of 500 µm (6 passes) was injected. Following the adipose tissue injection, a gentle massage was performed. 

### 2.5. Efficacy and Safety Measures

Both the volume loss and skin wrinkle severity of the temporal, malar, zygomatic, and jawline regions were assessed by the authors at baseline and 30, 90, and 150 days after the treatment. The volume and skin wrinkle severity were evaluated with a Canfield Vectra H1 3D imaging system (Canfield Scientific, Parsippany, NJ, USA), which is a validated tool for measuring both metrics [33]. Further, the subjective 5-point Global Aesthetics Improvement Scale (GAIS) was deployed and evaluations were performed by two board-certified esthetic plastic surgeons, FM (treating physician) and AG (blinded evaluator), practicing in Italy (Table 2). Complications were evaluated by FM and AG as they arose and were subsequently categorized into three tiers: severe, moderate, and minor. Following the tiering presented by Schiraldi et al., severe complications were considered life-endangering or permanently disabling, moderate complications were considered those requiring subsequent surgical intervention or correction, and minor complications were considered as those requiring a minor intervention or no intervention at all [34].

### 2.6. Statistical Analysis

The analysis of the GAIS data, which is ordinal, was carried out by comparing the average scaled scores at each time with grouped ANOVAs, a practice that is found acceptable in larger data sets that employ categorical data [35]. Significance is denoted as * *p* < 0.05, ** *p* < 0.01, *** *p* < 0.001, and **** *p* < 0.0001. 

## 3. Results

### 3.1. Demographics

A total of 149 female and 9 male patients (*n* = 158) enrolled in this study had maintained a stable weight, with variations not exceeding ±1 kg, in the five months both before and after the procedure (Table S1 in the Supplementary Materials). The BMIs ranged from 18.5 to 24.9. The average age for the female and male patients was 48.8 and 50.3, respectively. All the patients were Caucasian, with Fitzpatrick scores between 1 and 3. 

### 3.2. Injection Volumes

**Figure 2:**
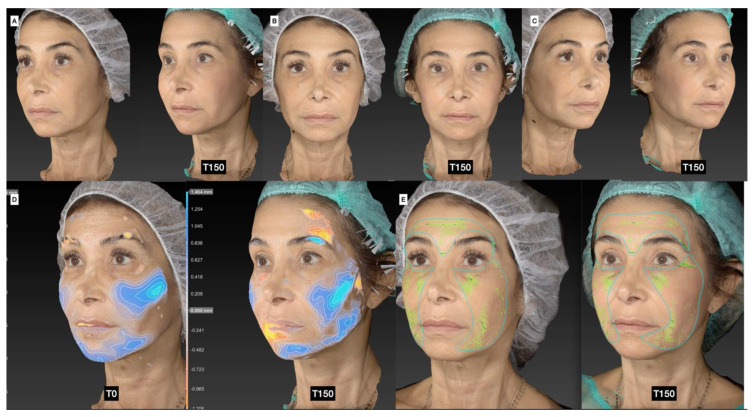
Three-dimensional scans of a 49-year-old female (**A**–**C**) pre-treatment (**left**) and 150 days after treatment (**right**) in the malar and zygomatic area and jawline, SEFFI 12 mL fluidified with 3 passages; CaHA 3 mL diluted 1:1. (**D**) Volumetric evaluation immediately post-treatment and 150 post-treatment. (**E**) Superficial wrinkles pre-treatment and 150 days post-treatment.

In this retrospective observational study, the authors evaluated the efficacy and complications of the novel treatment as well as complications in the donor site. Table 3 provides details on the volumes of CaHA and adipose tissue used, their respective dilutions/sizes, and the number of patients treated in the temporal, malar, zygomatic, and jawline regions. The average volumes of CaHA injected into the temporal, malar, zygomatic, and jawline areas were 0.8 mL, 1.5 mL, and 0.8 mL, respectively. For adipose tissue, the corresponding average volumes were 2.8 mL, 5.0 mL, and 3.7 mL. Most patients were administered with a 1:1 dilution of CaHA and 600 µm adipose tissue clusters. The three-dimensional scans of the patients can be found in Figure 2 and Figure S1 in the Supplementary Materials.

### 3.3. Enhancement in Skin Quality

Skin quality enhancement, as gauged by the Global Aesthetic Improvement Scale (GAIS) scores, revealed a progressive pattern of improvement. At the T30 mark, the treatment showed minimal and statistically non-significant enhancements. Specifically, the temporal area showed improvement in 8% of the cases. The malar and zygomatic areas combined had a 16.4% improvement rate (10.5% improved and 5.9% much improved). The jawline had a 13.5% total improvement rate (7.8% improved and 5.7% much improved).

By T90, there was a marked increase in skin quality. The temporal area had an 80.3% total improvement rate (72.3% improved, 4.4% much improved, and 3.6% very much improved). The malar and zygomatic areas combined reached an 83.5% total improvement rate (70.4% improved, 5.9% much improved, and 7.2% very much improved). The jawline saw an 84.5% total improvement rate (75.2% improved, 4.3% much improved, and 5.0% very much improved).

**Figure 3:**
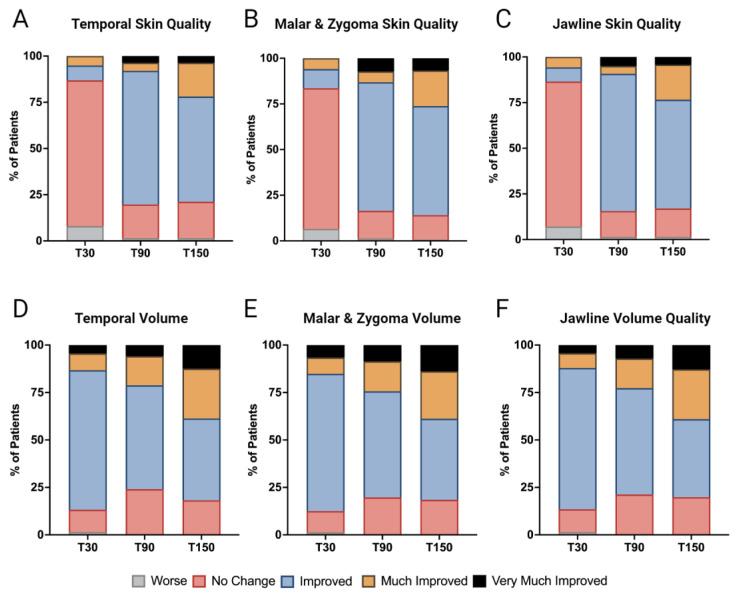
GAIS ratings for (**A**–**C**) skin quality and (**D**–**F**) volume in the temporal, malar and zygomatic, and jawline areas 30, 90, and 150 days after concurrent treatment with SEFFI and CaHA. T30 = 30 days post-treatment, T90 = 90 days post-treatment, and T150 = 150 days post-treatment.

At T150, while the improvement trend persisted, there was a slight dip. The temporal area had a 78.7% total improvement rate (56.9% improved, 18.2% much improved, and 3.6% very much improved). The malar and zygomatic areas combined had an 86.2% total improvement rate (59.9% improved, 19.7% much improved, and 6.6% very much improved). The jawline had an 83% total improvement rate (59.6% improved, 19.1% much improved, and 4.3% very much improved). Changes in the responder rates for the temporal, malar and zygomatic, and jawline skin quality, based on the GAIS scoring, are seen in Figure 3A–C and visualized by the skin quality assessments in Figure 3 and Figure S1 in the Supplementary Materials.

### 3.4. Volume Restoration

The evaluation of volume enhancement, as assessed through the analysis of Global Aesthetic Improvement Scale (GAIS) scores at various time points, yields compelling insights into the effectiveness of the combined treatment approach. Specifically, at the T30 time point, we observed a statistically significant increase in volume across the facial regions. The temporal region had an 86.9% total improvement rate (73.7% improved, 8.8% much improved, and 4.4% very much improved). Similarly, the malar and zygomatic areas combined reached an 87.6% total improvement rate (72.4% improved, 8.6% much improved, and 6.6% very much improved). The jawline also experienced noteworthy volume augmentation with a total improvement rate of 86.8% (74.5% improved, 7.8% much improved, and 4.3% reporting very much improved).

At T90, the volume enhancement remained considerable. The temporal area had a total improvement rate of 75.8% (54.7% improved, 15.3% much improved, and 5.8% very much improved). The malar and zygomatic areas had a combined total improvement rate of 80.3% (55.9% improved, 15.8% much improved, and 8.6% very much improved). The jawline maintained its improvement trend with an improvement rate of 78.7% (56% improved, 15.6% much improved, and 7.1% very much improved).

By T150, the volume remained significantly increased from the baseline. The temporal area had an improvement rate of 81.8% (43.1% improved, 26.3% much improved, and 12.4% very much improved). Similarly, the malar and zygomatic areas exhibited substantial volume enhancement, with a total improvement rate of 81.6% (42.8% improved, 25% much improved, and 13.8% very much improved). The jawline maintained had an improvement rate of 80.1% (41.1% improved, 26.2% much improved, and 12.8% much improved). Changes in the responder rates for the temporal, malar and zygomatic, and jawline skin quality, based on the GAIS scoring, are seen in Figure 3D–F and visualized by the skin quality assessments in Figure 3 and Figure S1 in the Supplementary Materials.

### 3.5. Photographic and Photogrammetric Results

**Figure 4:**
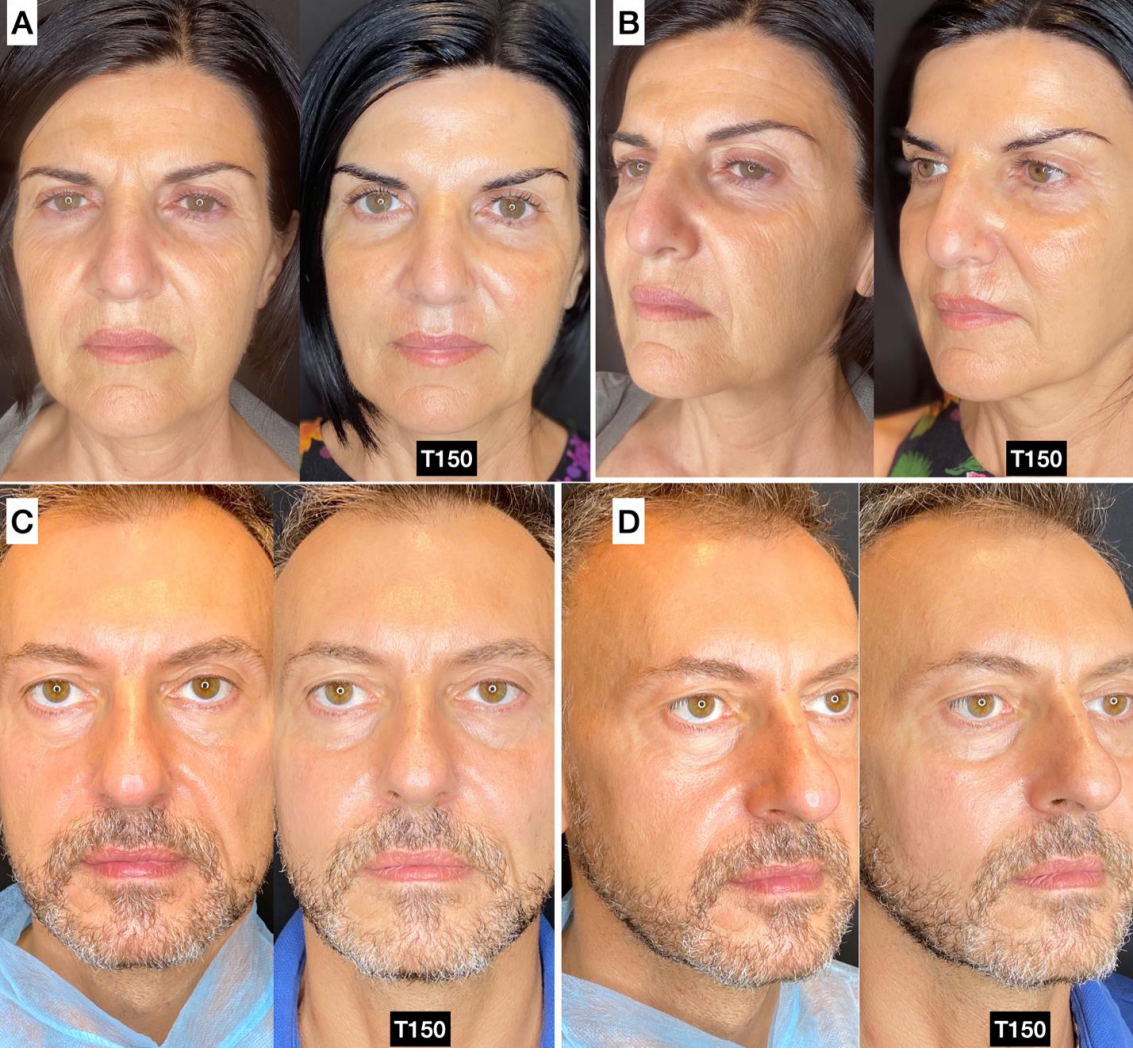
(**A**,**B**) A 51-year-old female before (left) and 150 days post-treatment (right) in the temporal, malar, and zygomatic area: guided SEFFI 20 mL fluidified with 3 passages; CaHA diluted 1:2. (**C**,**D**) A 46-year-old male before (left) and 150 days after (right) treatment in the malar area: guided SEFFI 14 mL fluidified with 3 passages; CaHA 1.5 mL diluted 1:1.

Both photographic and photogrammetric results in a variety of different patients can be seen in Figure 4 and Figure S2 in the Supplementary Materials. The 3D images show the redistribution and retention of volume in the face, while the identified rhytids decreased. Notably, minimal change at T30 was observed for most patients. The authors found the treated patients to retain a natural appearance with panfacial rejuvenation evident.

### 3.6. Complications

#### 3.6.1. Complications at Donor Sites

In addition to measuring efficacy, we sought to assess complications arising from both the donor and injection sites during the combined treatments using guided SEFFI and CaHA. In these cases, the abdomen was the primary donor site (52%), followed by the trochanteric region (32%), the hip (12.6%), the knee (2.8%), and other regions (0.6%). Complications were stratified into three tiers: severe, moderate, and minor. Our findings demonstrated an absence of severe complications at the donor site, underscoring the safety of the guided SEFFI procedure for adipose tissue harvesting. Moderate complications, necessitating subsequent interventions, include conditions such as fat hypertrophy, necrosis, cyst formation, skin irregularities, and asymmetry. Our research identified a singular occurrence of skin irregularity (0.6%) and one instance of asymmetry (0.6%). This suggests a minimal prevalence of moderate complications associated with the guided extraction of adipose tissue. Minor complications, which did not mandate secondary intervention, included prolonged edema, erythema, ecchymosis, telangiectasia, and acne reactivation. Within our patient group, 67 documented cases (42.4%) of ecchymosis at the donor site were noted, but all were resolved within one week. 

#### 3.6.2. Complications at Injection Sites

Regarding the complications at injection sites, the regions evaluated were the temporal, zygomatic, malar, and jawline areas. Consistent with the findings from the donor site, no severe or moderate complications were identified at the injection sites, which reaffirms the safety of the combined procedure. The predominant minor complication observed was ecchymosis, with 72 instances (45.5%) documented, all resolved within one week. Additionally, two cases (1.2%) of prolonged edema, persisting beyond three days, but resolved within ten days, were recorded. These data suggest that the complications at the injection sites were predominantly minor and manageable and that the concurrent treatment with SEFFI and CaHA appears safe.

## 4. Discussion

The rationale behind this procedure relies on foundational studies showing synergies between CaHA and adipose tissues. Regarding the fluidity of the guided SEFFI tissue and CaHA dilution, the decision to select the grade of fluidity of the guided SEFFI tissue and the dilution ratio of CaHA was guided by balancing volume augmentation and tissue biostimulation. On one hand, lower dilutions/larger SEFFI fragments result in superior volumization, but the increased fluidity of either component increases spread. Specifically, this rationale follows the principle that the greater the need for volume enhancement, the lower the fluidity of the guided SEFFI tissue should be. In practice, this translates to utilizing guided SEFFI with a thickness of 600 microns when a pronounced volume increase is required. Conversely, when the emphasis is on tissue regeneration, a higher fluidity guided SEFFI with a thickness of 500 microns is chosen. Moreover, the dilution ratio of CaHA is adjusted accordingly, with a 1:1 ratio preferred when volume is required and a 1:2 ratio chosen when tissue regeneration is the primary goal.

Regarding the different injection depths, the authors employ a strategic approach to the injection depth placement of each material. CaHA is injected slightly deeper, targeting the fibroblast-rich subcutaneous layer, while SEFFI tissue is placed in the more superficial subcutaneous layer, aligning with its regenerative potential and capacity to promote tissue remodeling.

A notable feature of this combined procedure is the ability to perform both treatments during the same session. This integrated approach minimizes treatment duration and patient inconvenience while capitalizing on the synergistic effects of tissue regeneration and volume enhancement. As both CaHA and SVF will promote tissue regeneration as well as neovascularization, the chances of survival of the transplanted adipose tissue may be improved. [36,37] Across all the regions treated, a progressive improvement in both the volume and skin quality was observed.

Interestingly, the efficacy of the treatments improved over time, with the later results showing gradual improvement. This observed gradual improvement can be explained by the lack of elastic moduli remaining in the diluted CaHA that would contribute to an immediate volumizing effect, and by the gradual nature of biostimulation [10]. For regenerative biostimulators like diluted CaHA and PLLA, volumization takes months to hit its peak effect, as the mechanisms of action are of a biological nature [38,39] Additionally, adipose transplantation also requires time for the grafts to revascularize and integrate with recipient tissue. This process consists of an acute regenerative adipogenic phase of fat graft survival that finishes around 3 months post-transplant followed by a chronic fat stabilization process that can persist for up to 9 months [40]. Taken together, the delayed peak effect in this combinatorial intervention is best explained by the delayed biostimulation and adipogenesis attributed to CaHA and SEFFI. Both the CaHA and SEFFI treatments, on their own, can drive volumetric correction and improvements in skin quality through direct filling and biological responses, though it is plausible their combined administration synergistically improves these effects.

Our observations highlight a consistent enhancement in skin quality over time and an improvement in volume across all the regions compared to the baseline. Both global volume and skin quality enhancements were significantly improved (reductions in the average GAIS scores) at each time point (Figure S3 in the Supplementary Materials). The photogrammetric and photographic results show improved volumization in the desired regions and skin quality improvements in both radiance and rhytids. Notably, this procedure was well tolerated, with few mild and no severe adverse events. 

## 5. Limitations

There exist several limitations in the current study. First, this retrospective observational study lacks control groups. Retrospective observational studies have limitations including rigorous experimental design that includes control groups. A three-armed study including a no-treatment control, a CaHA-only, and a SEFFI-only group delineating the impact of each treatment on each observed outcome (skin quality, volumization, GAIS, etc.) is thus warranted. Without comparison to a gold standard treatment, it is difficult to assess the efficacy of the combinatorial treatment. Additionally, this study employs only photogrammetric and photographic analyses that are subjectively evaluated using the 5-point GAIS scoring system. Deploying investigator GAIS, while a common practice, introduces the possibility of bias and persists as a non-empirical evaluation method. Therefore, quantifiable changes and enhanced analysis evaluating changes in the biophysical properties of the skin may further capture the synergy of combined CaHA and SEFFI.

## 6. Conclusions

The patients in this retrospective observational study showed improvements in skin quality and facial volume enhancement following concurrent treatment with SEFFI and CaHA. The data revealed an improvement in skin quality over time. Initial modest enhancements at T30 transitioned to substantial improvements by T90, especially in the temporal, malar, and zygomatic regions. Facial volume enhancement was noted across all the time points, with the most significant improvements at T30, and sustained progress extending to T150. Our findings highlight the potential of the combined guided SEFFI and CaHA in esthetic and regenerative medicine. This innovative protocol offers a novel treatment for skin aging. Additionally, no significant adverse events were captured. Further controlled research will be pivotal in expanding our understanding of this combined treatment’s potential in esthetic medicine.

## Figures and Tables

**Table 1:** SEFFI protocol for size reduction in tissue clusters.

Desired Cluster Dimension (µm)	Number of Passages	Cannula or Needle Size (Gauge, Inner Diameter in mm)
800	0	18, 0.83
600	2–3	20, 0.60
500	5–6	21, 0.51
400	10–11	22, 0.41
200	20–30	27, 0.21

**Table 2:** GAIS scoring criteria.

Score	GAIS Rating
1	Very much improved
2	Much improved
3	Improved
4	No change
5	Worse
6	Much worse

**Table 3:** Volumes of CaHA or adipose tissue injected in each area.

Region	Number Treated	Average CaHA Volume (ml/Side)	Number of Diluted (1:1) CaHA Treatments (Number, %)	Number of Hyperdiluted (1:2) CaHA Treatments (Number, %)	Average Guided SEFFI Volume (ml/side)	Number of 600 µmGuided SEFFI Treatments (Number, %)	Number of 500 µmGuided SEFFI Treatments (Number, %)
Temporal	117	0.8	89 (76.1%)	28 (23.9%)	2.8	101 (86.3%)	16 (13.7%)
Malar and Zygomatic	152	1.5	115 (75.7%)	37 (24.3%)	5	123 (80.9%)	29 (19.1%)
Jawline	113	0.8	82 (72.6%)	31 (27.4)	3.7	84 (74.3%)	29 (25.7%)

## Data Availability

Data are available from the authors upon reasonable request.

## Supplementary Materials

The following supporting information can be downloaded at: https://www.mdpi.com/article/10.3390/jcm13154381/s1, Table S1. Patient Demographics. Figure S1. A 49-year old female (A,C) pretreatment, (B,D) 90 days post treatment in the malar and zygomatic area with SEFFI (15 mL; fluidify 3 passages) and CaHA 1.5 ml diluted 1:1. (E–H) Volume evaluation from 0 to 150 days post treatment. (I–K) Superficial wrinkles at baseline and through 150 days following treatment. Figure S2. (A) Female 57 yrs old: pretreatment and 180 days post treatment: temporal malar, zygomatic area and periocular area: guidedSEFFI 30 mL fluidify 3 passages and 6 passages in periocular area, CaHA 3 mL diluted 1:1. (B) Female 46 yrs old: A pretreatment and 180 days post treatment: temporal malar, zygomatic area and periocular area: guidedSEFFI 28 mL fluidify 3 passages and 6 passages in periocular area, CaHA 3 mL diluted 1:1. (C) Female 58 yrs old: A pretreatment and 150 days post treatment: temporal malar, zygomatic area and periocular area: guidedSEFFI 25 mL fluidify 3 passages and 6 passages in periocular area, CaHA 4.5 mL diluted 1:1. (D–F) Female 49 yrs old: A pretreatment and 150 days post treatment: temporal malar, zygomatic area and periocular area: guidedSEFFI 22 mL fluidify 3 passages and 6 passages in periocular area, CaHA 1.5 mL diluted 1:1. Figure S3. Overall improvements in skin quality and volume based on the GAIS scale 30, 90, and 150 days after treatment. **** *p* < 0.0001.
