# Supplementary material for: Guided SEFFI and CaHA: A Retrospective Observational Study of an Innovative Protocol for Regenerative Aesthetics
Source: J Clin Med. 2024 Jul 26;13(15):4381. doi: 10.3390/jcm13154381 (PMC11313436; doi:10.3390/jcm13154381)
Supplement: Supplementary file 1 [file jcm-13-04381-s001.zip › jcm-3086840-supplementary.pdf]

Supporting Information

Guided SEFFI and CaHA: A Retrospective Observational Study of an Innovative Protocol for Regenerative Aesthetics

Fabrizio Melfa[1], Alec McCarthy[2], Shino Bay Aguilera[3], Jani van Loghem[4] , Alessandro Gennai[5]*

[1]Mediaging Clinic Center, Palermo, Italy

[2]Merz Aesthetics, Raleigh, NC, USA

[3]Shino Bay Cosmetic Dermatology & Laser Institute, Fort Lauderdale, FL, USA

[4]UMA Academy, Amsterdam, Netherlands

[5]Studio Gennai, Bologna, Italy

***Corresponding author**: agennai@mac.com

Keywords: SEFFI, SVF, CaHA, calcium hydroxylapatite, Radiesse, skin priming

Supplemental Table S1. Patient Demographics

Sex	Number of Patients	Age (average)	BMI (range)
Male	9	48.8	18.5-24.9
Female	149	50.3	

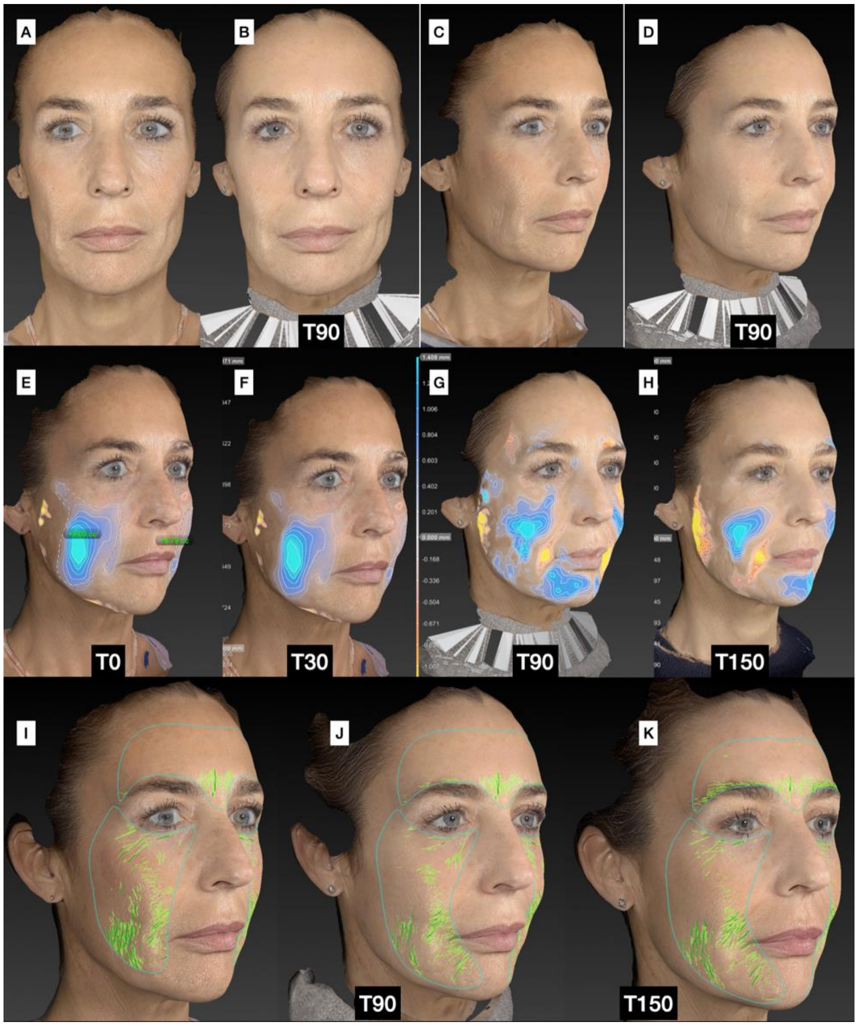

Figure S1. A 49-year old female (A,C) pretreatment, (B,D) 90 days post treatment in the malar and zygomatic area with SEFFI (15 ml; fluidify 3 passages) and CaHA 1.5 ml diluted 1:1. (E-H) Volume evaluation from 0 to 150 days post treatment. (I-K) Superficial wrinkles at baseline and through 150 days following treatment.

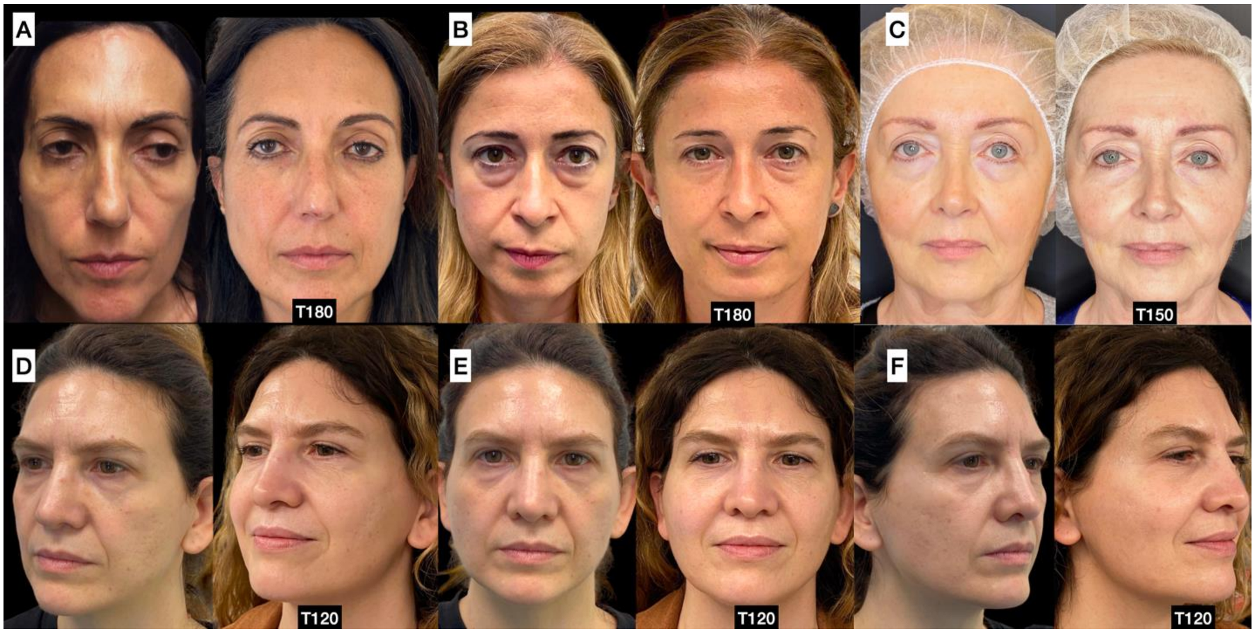

Figure S2. (A) Female 57yrs old : pretreatment and 180 days post treatment : temporal malar, zygomatic area and periocular area : guidedSEFFI 30ml fluidify 3 passages and 6 passages in periocular area , CaHA 3 ml diluted 1:1. (**B)** Female 46 yrs old : A pretreatment and 180 days post treatment : temporal malar, zygomatic area and periocular area : guidedSEFFI 28 ml fluidify 3 passages and 6 passages in periocular area , CaHA 3 ml diluted 1:1. (**C)** Female 58 yrs old : A pretreatment and 150 days post treatment : temporal malar, zygomatic area and periocular area : guidedSEFFI 25 ml fluidify 3 passages and 6 passages in periocular area , CaHA 4.5 ml diluted 1:1. (**D,E,F)** Female 49 yrs old : A pretreatment and 150 days post treatment : temporal malar, zygomatic area and periocular area : guidedSEFFI 22 ml fluidify 3 passages and 6 passages in periocular area , CaHA 1.5 ml diluted 1:1

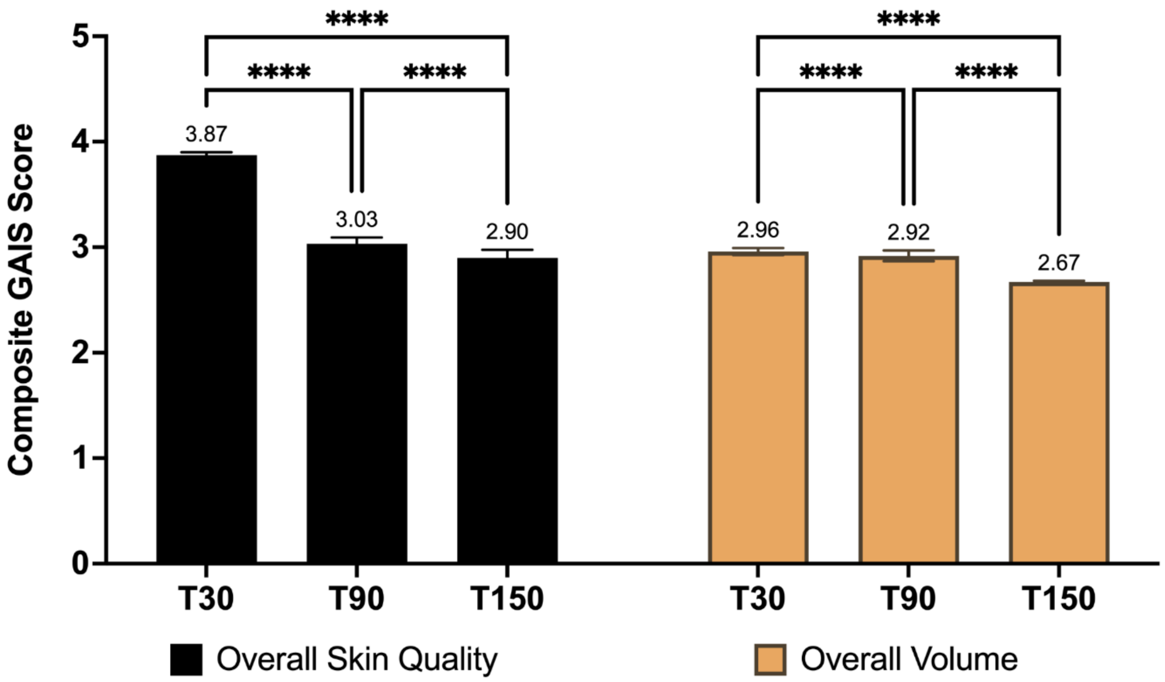

Figure S3. Overall improvements in skin quality and volume based on the GAIS scale 30, 90, and 150 days after treatment. ****P < 0.0001.
